# Association between maximum tongue pressure in healthy elderly individuals and demographic and dental characteristics

**DOI:** 10.1590/2317-1782/e20240153en

**Published:** 2025-02-10

**Authors:** Darley Luiz Gomes Ribeiro, Thais Mendes Rocha Alves Vieira, Aline Mansueto Mourão, Andréa Rodrigues Motta, Laelia Cristina Caseiro Vicente

**Affiliations:** Setor de Fonoaudiologia, Hospital Risoleta Tolentino Neves, Belo Horizonte (MG), Brasil.; 2 Departamento de Fonoaudiologia, Universidade Federal de Minas Gerais – UFMG - Belo Horizonte (MG), Brasil.

**Keywords:** Aged, Tongue, Pressure, Dentition, Periodontal Prosthesis, Oral Health

## Abstract

**Purpose:**

This study analyzed tongue pressure in healthy older adults during maximum voluntary contraction in the anterior and posterior regions and verified whether it was associated with sex, age, and dental status.

**Methods:**

This is a cross-sectional, observational, descriptive study with a non-probabilistic sample of 128 active and healthy older adults of both sexes. The evaluation consisted of a medical history survey to collect personal data, cognitive screening, and assessment of dental status and tongue pressure, using the Iowa Oral Performance Instrument. The anterior and posterior tongue pressures at maximum contraction were the response variables, and age, sex, number of natural teeth, and conditions related to dentures were the explanatory variables. The significance level was set at 5% for data analysis.

**Results:**

Males had greater anterior tongue pressure. The anterior and posterior tongue pressure measurements were significantly different between participants aged 60 to 69 years and those over 80 years. No significant differences were found regarding dental status and denture fitting.

**Conclusion:**

Anterior and posterior tongue pressure values were higher in men and decreased after the age of 80. The conditions related to the number of teeth and denture use and fitting did not influence the tongue pressure levels.

## INTRODUCTION

Population aging is one of the greatest challenges for public health. The term healthy aging refers not only to the absence of disease^(1)^. As proposed by the World Health Organization, it is the process of developing and maintaining functional capacity that allows well-being in old age. Thus, it has a broader concept, encompassing these people’s physical capacities and constant participation in society^(2)^.

Changes in the stomatognathic system caused by natural aging, called senescence, affect chewing, taste, and swallowing and can reduce food intake and consequently pose a risk of malnutrition, illness, and hospitalizations^(3)^.

The tongue is an important organ of the human body and actively participates in the stomatognathic functions of sucking, breathing, chewing, swallowing, and speaking^(4)^. It is composed of intrinsic muscles (without a bony insertion – superior longitudinal, inferior longitudinal, transverse, and vertical muscles) and extrinsic muscles (with a bony insertion – palatoglossus, genioglossus, hyoglossus, and styloglossus)^(4)^.

Changes in oral motor function can occur with aging due to the reduction in the size and strength of the striated muscles throughout the body. Thus, the tongue significantly reduces peak pressure with aging^(5)^.

Electronic equipment is used to measure tongue pressure. The best known is the Iowa Oral Performance Instrument (IOPI), which has been on the market since the 1990s. The JMS Tongue Pressure Measurement Device (launched in Japan in 2011 and expanding sales to the global market in 2022) and the Biofeedback Pró-Fono: Lip and Tongue Pressure (PLL Pró-Fono, developed in Brazil) provide pressure measures in kilopascals (kPa)^(6)^.

As mentioned, the tongue plays an important role in the biomechanics of swallowing. The literature indicates that the incidence of aspiration pneumonia and the decrease in swallowing function was greater in patients with oral fragility and reduced tongue pressure^(7)^.

Compromised oral conditions such as tooth loss, poorly fitted dentures, difficulty chewing, and xerostomia lead to problems in concentrating and manipulating the food bolus, which can result in voluntary swallowing adaptations, such as multiple swallows^(8)^. Thus, preserving oral health and functions in older adults, such as tongue pressure and number of teeth, can reduce the likelihood of their functional decline^(9,10)^.

Considering the fundamental role of the tongue in the stomatognathic system and older people’s deficient oral conditions and chewing, this study seeks to demonstrate the association between tongue pressure and demographic and dental characteristics. This may help develop targeted assessment and rehabilitation protocols for oral health promotion strategies among healthy older adults.

Therefore, this study aimed to analyze tongue pressure in healthy older adults during maximum voluntary contraction and verify whether it is associated with sex, age, and dental status.

## METHODS

This is a cross-sectional, observational, descriptive study. Data were collected at two locations: the Reference Center for Older People (CRPI) and the Risoleta Tolentino Neves Hospital (HRTN), both in Belo Horizonte, Brazil. The project was approved by the Research Ethics Committee of the Federal University of Minas Gerais (UFMG), under evaluation report number 1.799.909, and by the co-participating institution, the Belo Horizonte Municipal Health Department, under evaluation report number 1.830.525. All participants who agreed to participate signed an informed consent form.

The participant inclusion criteria were being 60 years or older; of either sex; not having any disease or history of neurological diseases, craniofacial malformation, cancer or sequelae of head and neck cancer treatment; and not having language impairment that would interfere with understanding the assessment. Those unable to perform all established procedures were excluded from the study.

Both the CRPI and the HRTN conducted an active search for older adults who met the eligibility criteria. The hospital invited the companions of hospitalized patients. These invited older adults initially underwent a brief assessment to verify whether they met the eligibility criteria, obtaining data on sex, date of birth, education, health history, and eating habits. Then, they underwent cognitive screening with the Mini-Mental State Examination (MMSE) test, using the cut-off point found in the literature for the Brazilian population, according to education level^(11)^.

Thus, the sample had 128 healthy older people, with a mean age of 70 years, a minimum of 60 years, and a maximum of 97 years (SD = 7.8). The sample size was adequate to obtain 80% statistical power in estimating older adults’ maximum tongue pressure. For this estimation, the bilateral Student's t-test was applied to a sample, considering a standard deviation of 13.58^(12)^ and a significance level of 0.05. The calculations were performed using Minitab 14 Release software.

The participants underwent individualized assessments for data collection at the respective locations where they were recruited, performed by two speech-language-hearing pathologists with experience in oral motor therapy, both of whom had been previously trained to apply the instruments used in this study. For interrater reliability analysis, 20% of the sample was assessed by both independently, and the Kappa coefficient test yielded a result of 96%, a high level of agreement^(13)^.

The older adults were assessed with the following: 1) dental status assessment, verifying the number of teeth, denture use, time of denture use, their fitting, and their last visit to the dentist. The dentures were considered well-fitted when they remained fixed to the upper and/or lower alveolar ridge as the mouth was opened. This study considered fixed dentures as natural teeth since they are stable and do not compromise the functions of the stomatognathic system^(14)^; 2) tongue assessment, measuring tongue pressure with the Iowa Oral Performance Instrument (IOPI), a portable instrument with an air-filled silicone bulb (approximately 3.5 cm long and 4.5 cm in diameter, with an internal volume of 2.8 ml), connected to a plastic tube (11.5 cm long). Its measurement is displayed on the LCD screen and the values ​​are expressed in Kilopascals (kPa)^(15)^.

For the IOPI assessment, participants sat on a chair at a 90° angle and were instructed to push the bulb positioned on the incisive papilla (anterior measurement) and on the hard palate (posterior measurement)^(16)^ as strongly as possible. Four 3-second measurements were collected, with a 60-second interval between them – the first was a training measurement and the others were analyzed. The highest pressure was considered the maximum measurement^(17)^.

Anterior and posterior tongue pressure at maximum contraction were the response variables in this study; and age, sex, number of natural teeth, type of dentition, and denture fitting were the explanatory variables.

The data were analyzed using the Statistical Package for the Social Sciences (SPSS), version 21.0, using measures of central tendency and dispersion to present continuous variables and frequency distribution for categorical variables. The t-test or ANOVA, Tukey test, and Spearman correlation were used for the explanatory variables. All analyses used a 5% significance level.

## RESULTS

The most frequent age range among the 128 healthy older participants was between 60 and 69 years, and the majority were females. Few older adults were toothless; the mean and median number of natural teeth were 13.28 and 11.00 (SD = 11.68), respectively, with a minimum of zero and a maximum of 32 teeth. Of those who used dentures (60.9%), the mean time of use was 20 years – although 25.6% of the sample did not know how long they had used their dentures. Most older people who used dentures were well-fitted, and a large part of the sample had had a dental appointment less than 1 year before (Table 1).

**Table 1:** Sociodemographic characteristics and dental status of healthy older people

**Characteristics**	**N**	**%**
**Sex**		
Females	98	76.6
Males	30	23.4
Total	128	100.0
**Education level**		
Illiterate	5	3.9
Middle school incomplete	56	43.8
Middle school graduate	18	14.1
High school incomplete	2	1.6
High school graduate	27	21.1
Bachelor’s degree	20	15.6
Total	128	100.0
**Age range (years)**		
60-69	66	51.6
70-79	48	37.5
80 or older	14	10.9
Total	128	100.0
**Number of teeth**		
Less than 11	65	50.8
More than 11	63	49.2
Total	128	100.0
**Dentition**		
Natural	46	35.9
Dentures	37	28.9
Natural and dentures	41	32.0
Toothless	4	3.1
Total	128	100.0
**Location of the dentures**		
Upper and lower	54	69.2
Upper	18	23.1
Lower	6	7.7
Total	78	100
**Denture fitting**		
Well-fitted	65	83.3
Poorly fitter	13	16.7
Total	78	100.0
**Time of denture use**		
Less than 20 years	33	42.3
More than 20 years	25	32.1
Unknown	20	25.6
Total	78	100.0
**Last visit to the dentist**		
Less than 1 year	61	47.7
1 year to 4 years and 9 months	29	22.6
5 years to 9 years and 9 months	16	12.5
More than 10 years	12	9.4
Unknown	10	7.8
Total	128	100.0

The mean anterior and posterior tongue pressure in healthy older adults was 40.8 kPa and 43.4 kPa, respectively. Males had higher anterior tongue pressure (p = 0.028). Regarding age, the mean anterior and posterior tongue pressure was significantly different between those aged 60 to 69 years and those over 80 years old – the tongue pressures decreased with advancing age. No significant differences were found regarding dental status and denture fitting, indicating an absence of association with the mean tongue pressure (Table 2).

**Table 2:** Comparison of mean anterior and posterior maximum tongue pressure with demographic variables and dental status

**Characteristics**	**Maximum tongue pressure** *					
	**Anterior**			**Posterior**		
	**Mean (kPa)**	**SD**	**p-value**	**Mean (kPa)**	**SD**	**p-value**
**Sex**						
Females	37.62	13.85	0.028*	41.21	12.86	0.095*
Males	44.00	13.41		45.60	11.09	
**Age (years)**						
60-69*	42.48	13.15	0.012**	44.23	12.63	0.050**
70-79	36.38	13.56		41.50	12.44	
80 or older*	32.64	15.64		35.43	10.68	
**Dentition**						
Natural	37.52	15.12	0.542**	39.76	12.09	0.107**
Dentures	38.95	14.49		43.59	12.75	
Natural + dentures	40.29	12.09		42.61	12.69	
Toothless	47.00	14.58		54.50	8.74	
**Denture fitting**						
Well-fitted	39.55	13.25	0.952*	42.32	12.58	0.552*
Poorly fitted	39.31	13.49		44.62	12.85	

* t-test;

** ANOVA test; SD = standard deviation

Spearman's correlation analyzed the association between the number of teeth and the mean anterior and posterior tongue pressure. The result showed a weak negative agreement with posterior tongue pressure – i.e., as the number of teeth increased, posterior tongue pressure decreased slightly. The correlation between the number of teeth and anterior tongue pressure was also inverse but with no difference (Table 3).

**Table 3:** Correlation between the number of teeth and maximum tongue pressure

**Mean maximum tongue pressure**	**Number of teeth**	
	**Spearman’s correlation**	**p-value**
**Anterior**	-0.103	0.255
**Posterior**	-0.203	0.023

## DISCUSSION

The results of this study revealed that the mean maximum anterior and posterior tongue pressure of healthy older adults was associated with sex and age. However, there was no association with their dental status, number of teeth, and denture use.

The mean anterior tongue pressure in this study was 40.8 kPa, and the posterior one was 43.4 kPa. Recent studies have shown similar values ​​for maximum anterior and posterior tongue pressure^(18,19)^. The anterior region of the tongue has a significant amount of connective and adipose tissue, with a predominance of type II fibers, which are known for their rapid contraction^(20)^. The posterior region has a greater number of type I muscle fibers, which have a slower but prolonged and more intense contraction^(20)^. Consequently, the posterior tongue pressure is less affected by aging than the anterior one^(16)^. This is due to the characteristics of the muscle fibers in this area and the fact that, with aging, the tongue undergoes muscular changes similar to the skeletal muscles of the body, including age-related muscle atrophy, a reduction in the number of muscle fibers, and an increase in the amount of fat^(16)^.

Moreover, men exerted significantly greater tongue pressure with the bulb in the anterior position than women, with a difference of approximately 8 kPa. This agrees with the literature, which highlights the tendency for generally more pronounced tongue muscle strength in men than in women due to the physical characteristics of the striated skeletal muscles, which are notably different in terms of power and efficiency^(18,21,22)^. On the other hand, the measurement with the bulb in the posterior position was not different between the sexes. This suggests that women can compensate for the lower power of their striated skeletal muscles by using other muscle groups – for instance, by using the tongue muscles to maintain the chewing power, naturally lower in women than in men^(18)^.

The literature shows that aging has an impact on muscle strength^(10,20)^ and tongue pressure decreases with advancing age, with a more significant reduction after the age of 80^(18,23)^. The present study had similar findings, as the maximum tongue pressure was lower in this age group than in those aged 60 to 69 years, with a difference of 10 kPa in the anterior and nine kPa in the posterior bulb position. This decrease in pressure is related to the loss of muscle mass and strength that occurs with aging (a consequence of sarcopenia), and may be an indicator of frailty in older people^(24)^. A systematic review with meta-analysis compared tongue pressure and handgrip strength in populations of different age groups; the results revealed higher values ​​in individuals under 60 years old than in those aged 60 years or older, with a mean value of 41.7 kPa^(25)^, similar to that found in the present study.

The lack of association between tongue pressure and dental status may be related to the compensatory mechanism of the tongue muscles, since tooth loss may require the tongue to work more intensely and for longer to compress food and form the bolus, which could potentially strengthen the tongue muscles in healthy adults^(4)^. Studies indicate that partially or totally toothless older adults have decreased tongue pressure values, evidencing reduced chewing and speech articulation capacity^(20,26)^. However, toothless participants in the present study had higher tongue pressure values ​​than those who had natural teeth or were using dentures. These data may be explained by the considerably fewer toothless older adults not rehabilitated with dentures, with only four individuals in the sample. Oral health care in Brazil is deficient, and access to information on oral hygiene is limited^(10)^. Although it is highly harmful to the individual, tooth loss is still considered a natural condition resulting from aging^(10)^.

The population analyzed in this study had a median of only 11 natural teeth, a number considered low. Dental studies generally indicate that the oral cavity must have at least 20 teeth for functional dentition, without needing dental prostheses^(27)^. The 2010 Brazilian epidemiological oral health survey found that 53.7% of older people aged 65 to 74 years in the five Brazilian macro-regions were completely toothless, placing the country at the top of the ranking when compared to European countries, India, China, and the United States^(28)^. However, this research did not find more recent publications on the topic. The lack of functional dentition can affect stomatognathic functions, mainly chewing and swallowing, leading people to adapt the food consistency. This can, in turn, compromise the intake and absorption of nutrients from the diet^(27)^.

In addition to the few natural teeth and the median 20 years of denture use, less than half of the sample sought follow-up with a dentist in the previous year. Professionals must monitor denture use frequently for adjustments, repairs, maintenance, and evaluation of soft tissues. This also helps to prevent diseases such as candidiasis and detect pre-malignant or cancerous lesions^(28)^. It is important to note that even older people who use dentures may face difficulties in chewing, as denture use requires regular monitoring by a dental professional^(10)^.

The imbalanced distribution between age ranges and sexes was identified as a limitation of this study. The maximum tongue pressure results were not affected by the number of teeth or denture fitting analysis. However, it is believed that the tongue can be adapted and reorganized, playing a more significant role in compressing food and forming the bolus in response to changes in oral health conditions^(4)^. It is recommended that future research use a stratified sample for a more accurate comparison of maximum tongue pressure in relation to natural teeth and dentures. In addition, it would be beneficial to include nutritional assessments to investigate the presence of sarcopenia. A recent study suggested a possible influence of dentofacial morphology in the differences in strength of the peri- and intraoral muscles – hence the importance of future studies thoroughly assessing orofacial structures^(29)^.

The data found in this study will enable speech-language-hearing promotion and prevention measures directed toward the specific needs of healthy older people, including those who use and do not use dentures.

## CONCLUSION

The mean maximum anterior and posterior tongue pressure in healthy older adults was 40.8 kPa and 43.4 kPa, respectively. These values ​​were higher in men and decreased after the age of 80, compared with those aged 60 to 69 years. Conditions related to the number of teeth, type of dentition, and denture fitting did not influence the levels of maximum tongue pressure.
