# Supplementary material for: Association between maximum tongue pressure in healthy elderly individuals and demographic and dental characteristics
Source: Codas. 2025 Feb 10;37(1):e20240153. doi: 10.1590/2317-1782/e20240153en (PMC11813070; doi:10.1590/2317-1782/e20240153en)
Supplement: Translation

# Translation: Associação entre pressão máxima de língua em idosos saudáveis e características demográficas e dentárias

**DOI:** 10.1590/2317-1782/e20240153pt

**Authors:** Darley Luiz Gomes Ribeiro, Thais Mendes Rocha Alves Vieira, Aline Mansueto Mourão, Andréa Rodrigues Motta, Laelia Cristina Caseiro Vicente

**Affiliations:** Setor de Fonoaudiologia, Hospital Risoleta Tolentino Neves, Belo Horizonte (MG), Brasil.; 2 Departamento de Fonoaudiologia, Universidade Federal de Minas Gerais – UFMG - Belo Horizonte (MG), Brasil.

**Keywords:** Idoso, Língua, Pressão, Dentição, Prótese Periodontal, Saúde Bucal

## Abstract

**Objetivo:**

O estudo analisou a pressão de língua em idosos saudáveis durante a contração voluntária máxima na porção anterior e posterior e verificou se houve associação com sexo, idade e condição dentária.

**Método:**

Trata-se de estudo transversal, observacional e descritivo com amostra não probabilística, composta por 128 idosos ativos e saudáveis, de ambos os sexos. A avaliação foi composta por anamnese para coleta de dados pessoais, rastreio cognitivo, avaliação da condição dentária e avaliação da pressão da língua por meio do Iowa Oral Performance Instrument. As variáveis resposta foram a pressão de língua em contração máxima (anterior e posterior) e as variáveis explicativas foram idade, sexo, número de dentes naturais e condições referentes às próteses dentárias. Foram considerados o nível de significância de 5% para análise dos dados.

**Resultados:**

Observou-se maior pressão anterior de língua no sexo masculino. Em relação à idade, a medida de pressão lingual tanto na região anterior quanto na posterior indicou diferença significativa entre os idosos de 60 a 69 anos comparados aos com mais de 80 anos. Quanto à condição dentária e à adaptação da prótese, não foram verificadas diferenças expressivas.

**Conclusão:**

Os valores de pressão de língua nas porções anterior e posterior mostraram-se mais elevados em homens e diminuíram após os 80 anos. As condições relacionadas ao número de dentes, presença e adaptação das próteses não influenciaram os níveis de pressão lingual.

## INTRODUÇÃO

O envelhecimento populacional é um dos maiores desafios para a saúde pública. Falar de envelhecimento saudável não engloba somente ausência de doença^(1)^. Como proposto pela Organização Mundial da Saúde o termo envelhecimento saudável é o processo de desenvolvimento e manutenção da capacidade funcional que permite o bem-estar em idade avançada, assim assume conceituação mais ampla, referindo-se não somente às suas capacidades físicas, mas à participação constante desses indivíduos dentro da sociedade^(2)^.

Mudanças no sistema estomatognático ocasionadas pelo envelhecimento natural, chamada senescência, repercutem na mastigação, no paladar e na deglutição, podendo reduzir a ingesta alimentar e consequentemente expor o indivíduo ao risco de desnutrição, adoecimento e internações hospitalares^(3)^.

A língua é um órgão importante do corpo humano e participa ativamente da realização das funções estomatognáticas de sucção, respiração, mastigação, deglutição e fala^(4)^. Ela é composta por músculos intrínsecos, aqueles que não possuem inserção óssea – longitudinal superior, longitudinal inferior, transverso e verticais – e músculos extrínsecos, que são aqueles que possuem inserção óssea – palatoglosso, genioglosso, hioglosso e estiloglosso^(4)^.

Alterações da função motora oral podem ocorrer com o envelhecimento devido à redução do tamanho e da força da musculatura estriada de todo o corpo, desta forma a língua diminui significativamente o pico pressórico com o envelhecimento^(5)^.

Para a medição da pressão de língua são utilizados equipamentos eletrônicos, sendo o mais conhecido o *Iowa Oral Performance Instrument* (IOPI) no mercado desde a década de 90. O “JMS *Tongue Pressure Measurement Device*” lançado no Japão em 2011, com expansão de vendas para o mercado global em 2022 e o Biofeedback Pró-Fono: Pressão de Lábios e de Língua (PLL Pró-Fono) desenvolvido no Brasil, sendo que todos trazem as medidas de pressão em quilopascal (kPa)^(6)^.

Como mencionado, a língua tem participação importante na biomecânica da deglutição, inclusive a literatura aponta que a incidência de pneumonia aspirativa e o decréscimo da função de deglutição foi maior em pacientes cuja pressão de língua estava diminuída e fragilidade oral instituída^(7)^.

O comprometimento das condições orais como a perda dentária, próteses mal adaptadas, dificuldade de mastigação e xerostomia acarretam problemas na concentração e manipulação do bolo alimentar, o que pode resultar em adaptações voluntárias de deglutição, como as deglutições múltiplas^(8)^. Assim, a conservação da saúde e das funções orais em idosos, como a pressão de língua e número de dentes, podem diminuir a probabilidade de declínio funcional nesta população^(9,10)^.

Considerando o papel fundamental da língua no sistema estomatognático, bem como as deficiências das condições orais e mastigação da população idosa, o presente estudo busca evidenciar a associação entre pressão de língua e as características demográficas e dentárias. Desta forma, é possível desenvolver protocolos de avaliação e reabilitação direcionados, a fim de promover estratégias de promoção da saúde oral em idosos saudáveis.

Frente ao exposto, o objetivo do presente estudo foi analisar a pressão de língua em idosos saudáveis durante a contração voluntária máxima e verificar se há associação com o sexo, idade e condição dentária.

## MÉTODO

Trata-se de estudo transversal, observacional e descritivo. A coleta de dados foi realizada em dois locais: Centro de Referência da Pessoa Idosa (CRPI) e Hospital Risoleta Tolentino Neves (HRTN), ambos em Belo Horizonte. O projeto foi aprovado no Comitê de Ética e Pesquisa da Universidade Federal de Minas Gerais (UFMG) sob o número do parecer 1.799.909 e pela Instituição Coparticipante Secretaria Municipal de Saúde de Belo Horizonte (SMSABH) sob o parecer 1.830.525. Todos os participantes que concordaram em participar assinaram o TCLE.

Os critérios de inclusão dos participantes foram ter idade igual ou superior a 60 anos; de ambos os sexos, não apresentar doenças ou história de doenças neurológicas, malformação craniofacial, câncer ou sequela do tratamento de câncer de cabeça e pescoço; não apresentar comprometimento de linguagem que interferissem na compreensão da avaliação. Foram excluídos do estudo aqueles que não conseguiram realizar todos os procedimentos estabelecidos.

Tanto CRPI como no HRTN foi realizada busca ativa dos idosos que preenchiam os critérios de elegibilidade, no hospital foram convidados os acompanhantes dos internados. Esses idosos convidados, inicialmente passaram por uma breve avaliação para verificar se preenchiam os critérios de elegibilidade. Para tanto, foram obtidos dados sobre sexo, data de nascimento, escolaridade, histórico de saúde e hábitos alimentares e depois realizado o rastreamento cognitivo por meio do teste Mini Exame do Estado Mental (MEEM) e utilizou-se o ponto de corte da literatura para a população brasileira, segundo a escolaridade^(11)^.

Assim, a amostra foi composta por 128 idosos saudáveis, com média de idade de 70 anos, com mínimo de 60 anos e máximo de 97 anos (DP=7,8). O tamanho amostral foi adequado para se obter 80% de poder estatístico na estimativa da pressão máxima da língua em indivíduos idosos. Para tal estimativa aplicou-se o teste bilateral *t-student* para uma amostra, considerando-se um desvio padrão de 13,58^(12)^ e um nível de significância de 0,05. Os cálculos foram feitos pelo *software Minitab 14 Release.*

Para coleta de dados, os idosos foram submetidos, nos respectivos locais onde foram recrutados, à avaliação individualizada, realizada por duas fonoaudiólogas com experiência em motricidade orofacial, sendo ambas treinadas previamente para aplicações dos instrumentos utilizados no presente estudo. Para análise de confiabilidade interavaliadores, 20% da amostra foi avaliada por ambas de forma independente e obteve-se, por meio do teste coeficiente *Kappa,* o resultado de 96%, valor de alta concordância^(13)^.

A avaliação com os idosos foi composta por 1) avaliação da condição dentária: verificou-se a quantidade de dentes, o uso de próteses dentárias, o tempo de uso das próteses, sua adaptação e quando foi a última consulta ao dentista. As próteses foram avaliadas como bem adaptadas, quando, ao abrir a boca, elas permaneciam fixas no rebordo alveolar superior e/ou inferior. Para esse estudo, as próteses fixas foram consideradas como dentes naturais, uma vez que são estáveis e não comprometem as funções do sistema estomatognático^(14)^; 2) avaliação da língua: para medir a pressão da língua foi utilizado o *Iowa Oral Performance Instrument* (IOPI), um instrumento portátil, constituído por um bulbo de silicone preenchido com ar (aproximadamente 3,5 cm de comprimento e 4,5 cm de diâmetro com um volume interno de 2,8 ml), conectado à um tubo plástico (11,5 cm de comprimento); sua medida é exibida na tela de LCD e os valores são expressos em Quilopascal (kPa)^(15)^.

Para a avaliação com o IOPI, os participantes estavam sentados em uma cadeira, na posição de 90°, foram orientados a empurrar o bulbo posicionado na papila palatina (medida anterior) e no palato duro (medida posterior)^(16)^ com a maior força possível. Foram coletadas quatro medidas com duração de três segundos cada, intervalo de 60 segundos entre elas: a primeira constitui a de treinamento e as demais foram analisadas. A maior pressão foi considerada como a máxima medida^(17)^.

As variáveis resposta consideradas no estudo foram a pressão de língua em contração máxima (anterior e posterior) e as variáveis explicativas foram: idade, sexo, número de dentes naturais, tipo de dentição e adaptação das próteses dentárias.

Os dados foram analisados no programa *Statistical Package for the Social Sciences* (SPSS), versão 21.0 e foram utilizadas medidas de tendência central e dispersão para apresentação das variáveis contínuas e de distribuição de frequência para as variáveis categóricas. Para as variáveis explicativas, foram utilizados os Teste-T ou Anova e teste de *Tukey* e a Correlação de *Spearman*. Para todas as análises foram considerados o nível de significância de 5%.

## RESULTADOS

Dos 128 idosos saudáveis pesquisados, a faixa de idade com maior frequência foi entre 60 e 69 anos e a maioria era do sexo feminino. Poucos idosos eram edêntulos, sendo que a média e a mediana do número de dentes naturais foi 13,28 e 11,00 (DP=11,68) respectivamente, com mínimo de zero e máximo de 32 dentes. Dos que usavam prótese dentária (60,9%), o tempo médio de uso foi de 20 anos. Ressalta-se que 25,6% da amostra não soube informar sobre o tempo de uso das próteses. A maioria dos idosos que utiliza próteses estavam bem adaptadas e grande parte da amostra realizou consulta odontológica há menos de 1 ano (Tabela 1).

**Tabela 1:** Características sociodemográficas e condições dentárias dos idosos saudáveis

**Características**	**N**	**%**
**Sexo**		
Feminino	98	76,6
Masculino	30	23,4
Total	128	100,0
**Escolaridade**		
Analfabeto	5	3,9
Fundamental incompleto	56	43,8
Fundamental completo	18	14,1
Médio incompleto	2	1,6
Médio completo	27	21,1
Superior completo	20	15,6
Total	128	100,0
**Faixa Etária (anos)**		
60-69	66	51,6
70-79	48	37,5
80 ou mais	14	10,9
Total	128	100,0
**Número de dentes**		
Menos de 11	65	50,8
Mais de 11	63	49,2
Total	128	100,0
**Dentição**		
Natural	46	35,9
Próteses	37	28,9
Natural e próteses	41	32,0
Edêntulo	4	3,1
Total	128	100,0
**Localização da prótese**		
Superior e inferior	54	69,2
Superior	18	23,1
Inferior	6	7,7
Total	78	100
**Adaptação das próteses**		
Bem adaptada	65	83,3
Mal adaptada	13	16,7
Total	78	100.0
**Tempo de uso das próteses**		
Menos de 20 anos	33	42,3
Mais de 20 anos	25	32,1
Não sabe informar	20	25,6
Total	78	100,0
Última consulta ao dentista		
Menos de 1 ano	61	47,7
De 1 ano a 4 anos e 9 meses	29	22,6
De 5 anos a 9 anos e 9 meses	16	12,5
Mais de 10 anos	12	9,4
Não sabe informar	10	7,8
Total	128	100,0

A média da pressão na língua anterior e posterior em idosos saudáveis foi de 40,8 kPa e 43,4 kPa, respectivamente. Observou-se maior pressão anterior de língua no sexo masculino (p=0,028). Em relação à idade, as médias de pressão lingual, tanto na região anterior quanto na posterior, indicaram diferença significativa entre os idosos de 60 a 69 anos comparados aos com mais de 80 anos, sendo as pressões de língua menores com o avançar da idade. Quanto à condição dentária e à adaptação da prótese, não foram obtidas diferenças expressivas, indicando ausência de associação com as médias de pressão lingual (Tabela 2).

**Tabela 2:** Comparação das médias da pressão máxima anterior e posterior da língua com as variáveis demográficas e condições dentárias

**Características**	Pressão máxima da língua*					
	**Anterior**			**Posterior**		
	**Média (kPa)**	**D.P.**	**Valor de p**	**Média (kPa)**	**D.P.**	**Valor de p**
**Sexo**						
Feminino	37,62	13,85	0,028*	41,21	12,86	0,095*
Masculino	44,00	13,41		45,60	11,09	
**Idade (anos)**						
60-69*	42,48	13,15	0,012**	44,23	12,63	0,050**
70-79	36,38	13,56		41,50	12,44	
80 ou mais*	32,64	15,64		35,43	10,68	
**Dentição**						
Natural	37,52	15,12	0,542**	39,76	12,09	0,107**
Próteses	38,95	14,49		43,59	12,75	
Natural + prótese	40,29	12,09		42,61	12,69	
Edêntulo	47,00	14,58		54,50	8,74	
**Adaptação das próteses**						
Bem adaptada	39,55	13,25	0,952*	42,32	12,58	0,552*
Mal adaptada	39,31	13,49		44,62	12,85	

* Teste T;

** Teste ANOVA; DP = Desvio Padrão

A associação entre o número de dentes e as médias das pressões anterior e posterior da língua foi analisada pela Correlação de *Spearman*. O resultado observado na pressão posterior da língua foi concordância fraca e negativa, isto é, à medida que aumenta o número de dentes, diminui levemente a pressão posterior da língua. A correlação entre o número de dentes e a pressão anterior da língua também foi inversa, porém não houve diferença (Tabela 3).

**Tabela 3:** Correlação entre o número de dentes e as pressões máxima da língua

**Média da pressão máxima da língua**	**Número de dentes**	
	**Correlação de Spearman**	**Valor-p**
Anterior	-0,103	0,255
Posterior	-0,203	0,023

## DISCUSSÃO

Os resultados do presente estudo revelaram associação entre as médias de pressão máxima de língua (anterior e posterior) de idosos saudáveis com o sexo e a idade. Todavia, ausência de associação em relação às condições dentárias, número de dentes presentes e uso de próteses dentárias de idosos saudáveis.

As médias de pressão lingual encontradas no presente estudo na porção anterior da língua foram de 40,8 kPa, enquanto na região posterior foram de 43,4 kPa. Estudos recentes mostram valores semelhantes para pressão máxima anterior e posterior de língua^(18,19)^. Na região anterior da língua, há uma quantidade expressiva de tecido conjuntivo e adiposo, com uma predominância de fibras do tipo II, que são conhecidas por sua contração rápida^(20)^. Já na região posterior, encontramos um maior número de fibras musculares do tipo I, que têm uma contração mais lenta, porém prolongada e mais intensa^(20)^. Consequentemente, a pressão exercida pela língua na parte posterior é menos afetada pelo envelhecimento em comparação com a porção anterior^(16)^. Isso se deve às características das fibras musculares nessa área e ao fato de que, com o envelhecimento, a língua passa por mudanças musculares de maneira semelhante aos músculos esqueléticos do corpo, incluindo a atrofia muscular relacionada à idade, uma redução no número de fibras musculares e um aumento na quantidade de gordura^(16)^.

Em relação ao sexo, a pressão exercida pela língua com o bulbo na posição anterior foi significativamente maior nos homens em comparação as mulheres, com diferença de cerca de 8 kPa. Essa observação coincide com a literatura, que ressalta a tendência de força muscular lingual geralmente mais pronunciada em homens do que em mulheres, devido às características físicas da musculatura estriada esquelética, que demonstram uma notável diferença em termos de potência e eficiência^(18,21,22)^. Por outro lado, a medição com o bulbo na posição posterior não revelou diferença entre os sexos. Isso sugere que as mulheres podem compensar a menor potência de sua musculatura estriada esquelética utilizando outros grupos musculares para auxiliar, como por exemplo o uso dos músculos da língua para manter o nível de potência de mastigação, por exemplo, que é naturalmente inferior nas mulheres em comparação com os homens^(18)^.

A literatura demonstra que o envelhecimento tem impacto na força muscular^(10,20)^, a pressão lingual diminui à medida que a idade avança, no entanto, os estudos indicam redução mais significativa a partir dos 80 anos^(18,23)^, como também foi observado no presente estudo. A pressão máxima da língua nessa faixa etária foi inferior à encontrada nos idosos com idades entre 60 e 69 anos, com uma diferença de dez kPa na posição anterior e nove kPa na posição posterior do bulbo. Essa diminuição na pressão está relacionada à perda de massa e força muscular que ocorre com o envelhecimento, uma consequência da sarcopenia, e pode ser um indicador de fragilidade em idosos^(24)^. Uma revisão sistemática com meta-análise comparou a pressão da língua e a força de preensão manual em populações de diferentes faixas etárias, os resultados revelaram valores mais elevados em indivíduos com menos de 60 anos, em comparação com aqueles com 60 anos ou mais, com valor médio de 41,7 kPa^(25)^, valor semelhante ao encontrado no presente estudo.

A ausência de associação entre a pressão de língua e a condição dentária pode estar relacionada ao mecanismo compensatório dos músculos da língua, uma vez que a perda de dentes pode requerer que a língua trabalhe de forma mais intensa e prolongada para comprimir os alimentos e formar o bolo alimentar, o que poderia potencialmente levar ao fortalecimento dos músculos da língua em adultos saudáveis^(4)^. Estudos apontam que idosos que são edêntulos parciais ou totais, observam-se valores diminuídos de pressão na língua, evidenciando redução da capacidade de mastigação e fonoarticulação^(20,26)^. No entanto, no presente estudo, os idosos edêntulos exibiram valores de pressão na língua mais elevados quando comparados aos idosos que possuíam dentes naturais ou estavam usando próteses dentárias. Acredita-se que esses dados possam ser explicados pelo fato de que o número de idosos edêntulos não reabilitados por próteses dentárias era consideravelmente menor, com apenas quatro indivíduos na população estudada. No Brasil, os cuidados com a saúde oral são deficientes, e o acesso a informações sobre higiene bucal é limitado^(10)^. Embora seja altamente prejudicial para o indivíduo, a perda dentária ainda é considerada como uma condição natural decorrente do envelhecimento^(10)^.

A população analisada neste estudo tinha mediana de apenas 11 dentes naturais, um número considerado baixo. Estudos odontológicos geralmente indicam que é necessário ter no mínimo 20 dentes na cavidade oral para se ter uma dentição funcional, sem a necessidade de próteses dentárias^(27)^. No levantamento epidemiológico de saúde bucal realizado em 2010 no Brasil, foi constatado que 53,7% dos idosos com idades entre 65 e 74 anos, nas cinco macrorregiões brasileiras, eram completamente edêntulos, colocando o país no topo do ranking se comparado a países europeus, Índia, China e Estados Unidos^(28)^. Não foram encontradas publicações mais atuais relacionadas ao tema. A falta de dentição funcional pode afetar as funções estomatognáticas, principalmente a mastigação e a deglutição, levando as pessoas a fazerem adaptações em relação à consistência dos alimentos. Isso pode, por sua vez, comprometer a ingestão e a absorção de nutrientes da dieta^(27)^.

Além do baixo número de dentes naturais e de mediana de 20 anos no tempo de uso de próteses, menos de metade da amostra procurou acompanhamento com um odontólogo no último ano. O uso de próteses dentárias exige acompanhamento frequente de um profissional para ajustes, reparos, manutenção e avaliação dos tecidos moles. Ademais, isso ajuda a prevenir doenças como candidíase e detectar lesões pré-malignas ou cancerígenas^(28)^. É importante observar que mesmo os idosos que fazem uso de próteses dentárias podem enfrentar dificuldades na mastigação, uma vez que o uso dessas próteses requer acompanhamento regular por um profissional odontólogo^(10)^.

A falta de distribuição equilibrada entre as diferentes faixas etárias e entre os sexos foi identificada como uma limitação deste estudo. Os resultados das medições da pressão máxima na língua não foram afetados pelo número de dentes presentes ou pela análise de adaptação das próteses dentárias. Entretanto, acredita-se que a língua tem a capacidade de se adaptar e reorganizar e desempenhe um papel mais significativo, a fim de comprimir os alimentos e formar o bolo alimentar em resposta a mudanças nas condições de saúde oral^(4)^. Para futuras pesquisas, é recomendável utilizar amostra estratificada com o objetivo de possibilitar comparação mais precisa entre a pressão máxima da língua em relação aos dentes naturais e às próteses. Além disso, seria benéfico incluir avaliações nutricionais para investigar a presença de sarcopenia. Em um estudo recente, foi sugerida uma possível influência da morfologia dentofacial nas diferenças observadas na força dos músculos peri e intraorais, daí a importância de estudos futuros que contemplem uma avaliação minuciosa das estruturas orofaciais^(29)^.

Com base nos dados encontrados, será possível desenvolver medidas de promoção e prevenção no campo da Fonoaudiologia que sejam direcionadas para as necessidades específicas de idosos saudáveis, tanto aqueles que usam próteses dentárias como os que não as utilizam.

## CONCLUSÃO

A média da pressão máxima na língua anterior e posterior em idosos saudáveis foi de 40,8 kPa e 43,4 kPa, respectivamente. Esses valores mostraram-se mais elevados em homens e diminuíram após os 80 anos, em comparação com idosos entre 60 e 69 anos. As condições relacionadas ao número de dentes, tipo de dentição e adaptação das próteses não influenciaram os níveis de pressão máxima da língua.
